# Three-dimensional easy morphological (3-DEMO) classification of scoliosis, part I

**DOI:** 10.1186/1748-7161-1-20

**Published:** 2006-12-05

**Authors:** Stefano Negrini, Alberto Negrini, Salvatore Atanasio, Giorgio C Santambrogio

**Affiliations:** 1 ISICO (Italian Scientific Spine Institute) Milan, Italy; 2 Fondazione Don Carlo Gnocchi IRCCS-ONLUS, Milan, Italy; 3 Department of Bioengineering, Polytechnic of Milan, Italy

## Abstract

**Background:**

While scoliosis has, for a long time, been defined as a three-dimensional (3D) deformity, morphological classifications are confined to the two dimensions of radiographic assessments. The actually existing 3-D classification proposals have been developed in research laboratories and appear difficult to be understood by clinicians.

**Aim of the study:**

The aim of this study was to use the results of a 3D evaluation to obtain a simple and clinically oriented morphological classification (3-DEMO) that might make it possible to distinguish among different populations of scoliotic patients.

**Method:**

We used a large database of evaluations obtained through an optoelectronic system (AUSCAN) that gives a 3D reconstruction of the spine. The horizontal view was used, with a spinal reference system (Top View). An expert clinician evaluated the morphological reconstruction of 149 pathological spines in order to find parameters that could be used for classificatory ends. These were verified in a mathematical way and through computer simulations: some parameters had to be excluded. Pathological data were compared with those of 20 normal volunteers.

**Results:**

We found three classificatory parameters, which are fully described and discussed in this paper: Direction, the angle between spinal pathological and normal AP axis; Shift, the co-ordinates of the barycentre of the Top View ; Phase, the parameter describing the spatial evolution of the curve. Using these parameters it was possible to distinguish normal and pathological spines, to classify our population and to differentiate scoliotic patients with identical AP classification but different 3D behaviors.

**Conclusion:**

The 3-DEMO classification offers a new and simple way of viewing the spine through an auxiliary plane using a spinal reference system. Further studies are currently under way to compare this new system with the existing 3-D classifications, to obtain it using everyday clinical and x-rays data, and to develop a triage for clinical use.

## Background

The third dimension has now become a real entity in the clinical study of scoliosis, but today the evaluation of the third dimension always requires, together with the use of radiographic projections, a wealth of clinical experience and considerable powers of abstraction in order to form an idea of what might be the real 3D behavior of the pathological spine under examination. The development of new technologies has somewhat reduced the restrictions mentioned above, especially in the research sphere [1-5]. However we are still far from achieving a concrete, reproducible picture and useful understanding of scoliosis as a 3D phenomenon. One of the main obstacles, due to the difficulty of visualization [6], is the inability to achieve a clinically useful representation of the deformity. A further problem, directly related to the first, is that both communication and comprehension are rendered difficult by the lack of a relevant codification of (and thus of the capacity to describe) the third dimension. Until these obstacles are removed, the use of the third dimension of scoliosis will continue to be confined to the sphere of research.

Since the first classification of scoliosis, proposed by Schulthess [7] and later refined by Ponseti [8], new ones have emerged: King [9,10], Coonrad [11], and Lenke [12-14] proposed new classifications mainly from therapeutic (surgical) points of view, even though progressively and roughly approaching also the 3D concept, including the sagittal profile together with the classical coronal one. Since our first presentation of this classification in 1996 [15-18] and 1999 [19,20], Poncet's group first proposed a possible 3D classification in 1998 [21] and then published it in 2001 on Spine [22], just like Duong [23] who recently proposed Fuzzy Clustering as a way to obtain it: but both these proposals are complex to understand and to visualize, and are derived from bioengineering studies more than from clinical evaluations of 3D results.

Stokes and the Scoliosis Research Society Working Group on 3D Terminology of Spinal Deformity [6] made the following assertion: "Visualization of anything three-dimensional is a great challenge. The approach we adopted attempts to accommodate this human limitation by making extensive use of the "auxiliary" planes on to which the spine is projected. Such measurements are not truly 3D, but this approach of using "quasi-3D" measurements represents a reasonable compromise between mathematical purity and conceptual and practical limitations". On the basis of these observations, facing the problem of looking and classifying a 3D object like the pathological spine, we decided to focus on a "quasi-3D" auxiliary plane like the Top View, that is a combination of the two classical AP and LL projections that allows a different and new view, which will be presented in this study. With the classic radiographic examination, the Top View is possible, but does nothing to further the understanding of spine behavior; in the literature, the Top View has already been described on the basis both of computerized reconstruction derived from conventional planar radiographic information [24-27] or from stereo-radiographs [1,2], and of examinations carried out by means of a surface analysis [28-30]; however, to our knowledge, nobody has so far attempted to formulate a new classification of spinal deformities on this basis.

In this study, we set out to develop a 3D codification of spinal deformities on the basis of their visualization through the Top View generated by one computerized non-invasive device. We used such a source of data because we needed a high number of 3D curves totally mathematically described to look at, already stored in a large database with the corresponding clinical and x-rays measurements, to develop and verify the possibility of defining a new classification. Nevertheless, our ultimate aim is, in order to further the understanding of third dimension complexity, to develop a 3D classification which is accessible to clinicians and which differs from the usual radiographic projections. With this aim, this paper and those immediately following in Part II and III, are only the first steps to verify the practicability of this idea, but the final objective already underway, is to obtain this classification from everyday usage tools like plain x-rays and clinical measurements.

## Materials and methods

### Population

We studied 149 (110 females) patients affected by adolescent idiopathic scoliosis (122), hyperkyphosis (23), or both (4) admitted for treatment to one of our institutes (FDCG) between January 1990 and January 1996. This is a referral clinic collecting patients from all over the country with high degree scoliosis both at first diagnosis or already treated without any success. The mean age was 16.3 ± 2.8 (range 12–20), weight and height were 53.0 ± 13.7 and 162.0 ± 9.8 respectively. Table 1 gives the radiographic characteristics of the patients; 63.8% of patients have not been treated before. We compared these data with a normal convenience sample. This included 16 females and 4 males, with a mean age of 14.6 ± 2.0 (range 12–19), weight and height were 49.9 ± 10.0 and 160.7 ± 13.1 respectively. All participants had no previous history of significant pathologies, but it was not possible to do radiographic exams to exclude minimal spinal deformities: for this reasons, we excluded 2 persons from the original sample of 22, whose AUSCAN Cobb degrees [31] were higher than 10° in the frontal and 50° in the sagittal plane.

**Table 1:** Radiographic data of studied population.

		Main curve(s) (mean ± S.D.)	Secondary curve (mean ± S.D.)
Double curves	90	37.9 ± 12.8 37.0 ± 11.6	
Single curves	30	37.6 ± 14	15.5 ± 21.7
Triple curves	4	24 ± 16.5 36.7 ± 18.5 27.7 ± 10.3	
Hyperkyphosis	26	Kyphosis: 65 ± 8.2 Lordosis: 58.6 ± 10.7	

### The AUSCAN System

All participants underwent an optoelectronic surface examination using the AUSCAN System (AUtomatic SColiosis ANalyser) [31], which is an automatic optoelectronic device specifically developed for the postural and functional analysis of patients affected by spinal deformities. The system is designed to compute in real-time, with a sampling rate of 100 Hz, the three-dimensional co-ordinates of a series of markers previously positioned on the skin of the analyzed subject. The basic components of the system [31] are two pairs of CCD TV-cameras, a FPSR (Fast Processor for Shape Recognition) image processor and a specially developed software package for data processing. The followed procedure includes a phase in which 27 passive skin markers (hemispheric shape, diameter 1 cm) are positioned on predetermined anatomical body landmarks: 19 on the posterior side of the patient and 8 on the anterior side. The reconstruction of the co-ordinates of the 10 markers, placed on the spinous processes (identified by palpation) of every second vertebra from C7 to S1, provides detailed data on spinal morphology. The relationship between the position of the skin markers and the centroids of the vertebrae has already been described in several studies [1,2]. The precision of the marker co-ordinates computed by the AUSCAN System has been studied by means of specific trials [30]: the experimental margin of error is less than 1 mm. The data are acquired with the patient standing for one second: computation is then based on the mean position of the markers during the acquisition. All patients were evaluated twice, while normal sample three times.

### The Top View by means of the AUSCAN System

We calculated the Top View using two different reference systems [6]:

• global: the spine is projected on to the horizontal plane, orthogonal to a reference vertical axis which corresponds to the line of gravity;

• spinal: the spine is projected on to the horizontal plane, orthogonal to a spinal vertical line that we identified in our study as the line linking the landmarks of the spinal processes of C7 and S1 (Stokes suggests D1 as a possible alternative to C7) [6].

**Figure 1:**
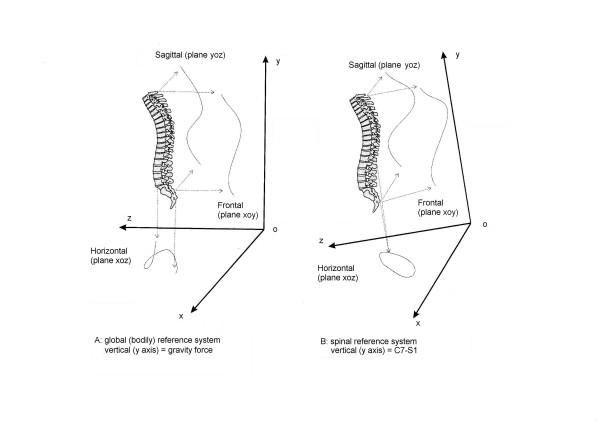
3D representation of a real pathological spine (right thoracic, left lumbar scoliosis). In this figure the projections of the spine in the three spatial planes is reported: the frontal (xoy) plane is usually seen in the AP radiographs, the sagittal (yoz) is that of the classical LL x-rays, while the horizontal (yoz) plane (Top View) is not usually considered and it is the one studied here. The Top View doesn't allow to see the effect of the y axis, but joins together the sagittal and frontal plane deviations: in this respect it represents a useful auxiliary plane to have a quasi-3D projection of the spine. The Top View can be seen in a global (bodily) reference system (on the left: A) in which the vertical (y) axis is the gravity line, or in a spinal reference system (on the right: B) in which the vertical (y) axis is the line joining C7 and S1. In this last situation, that is the one that proved to be useful and it is adopted throughout this study, the entire reference system rotates with respect to the gravity line, as it can be seen on phthe right (B). These figures refer to the same single subject: note the differences between global (A) and spinal (B) Top Views.

Figure 1 compares the spinal and the global Top Views computed in the same subject. The Top Views were obtained by means of the following procedure. The curve of the spine is reconstructed by interpolating the 10 markers placed on the spinal apophyses. The interpolation is carried out by calculating the cubic splines for each group of three consecutive markers in the frontal (8 splines: y_i _= f_i_(x), i = 1..8) and in the sagittal planes (8 splines: y_j _= f_j_(z), j = 1..8). The curves are sampled by extracting 100 points which are equidistant in relation to the vertical co-ordinate in the reference system ((x_i_, y_i_), i = 1..100; (z_j_, y_j_), j = 1..100). By projecting the points on to the plane, orthogonal to the vertical axis adopted, the 100 points forming the Top View ((x_i_, z_j_), i = j) are obtained. The sampling of the curve projected on to the plane, perpendicular to the vertical axis of the chosen reference system, is then performed in relation to the vertical co-ordinate: this justifies the definition of "quasi-3D" curve. The final curve is formed by a linear interpolation of pairs of consecutive points. In case of adoption of the spinal reference system, the first step is to rotate the spine until the vertical (global) and spinal (C7-S1) axes coincide (Figure 1).

### Classification methodology

On the basis of the Top View traces of all patients in both the global and spinal reference systems, classification criteria were developed by one of the authors (SN), experienced clinician, who was blinded to the patients' clinical-radiographic data. The aim was to identify the existence of any typical morphological feature that might allow grouping and comparison of the curves. The global Top View (Figure 1A) did not allow the curves to be grouped in a reasonable fashion on the basis of their morphology and was eliminated from any further computation. Thus, from now on, the term Top View only refers to the spinal Top View (Figure 1B). Obtained results were then reviewed by another author (AN) not having a clinical background. This was followed by a joint evaluation. Throughout this process, particular care was taken to eliminate any information deemed redundant, especially that deducible from the classic projections in the frontal and sagittal planes. The next phase was to identify the mathematical expression of the recognized morphological features, so as to make it possible to compute all considered parameters. In this paper we present a percentage, relative to the distribution in our population of the identified classificatory options, that has been defined on the obtained normative data. Then, as documented in the second part of this study [32], we verified the repeatability of obtained information. The last two phases of this study, which are to be reported in the third and fourth parts, focus on the comparison with classic radiographic classifications (Ponseti [8], King [9,10], Lenke [12-14]) and then with the existing 3D classification by Poncet [21,23].

The classification has been named 3-DEMO, the acronym of Three-Dimensional, Easy, Morphological classification, to summarize its characteristics. In fact, even if in this paper the Classification has been necessarily derived at first through a complex optoelectronic device, the aim was to find 3D morphological parameters easy to be understood by clinicians. This required the use of real 3D reconstructions of the spine. The following steps will include the development of means to obtain (and then use) this classification in everyday clinics.

### Graphical representation of the spinal Top View

**Figure 2:**
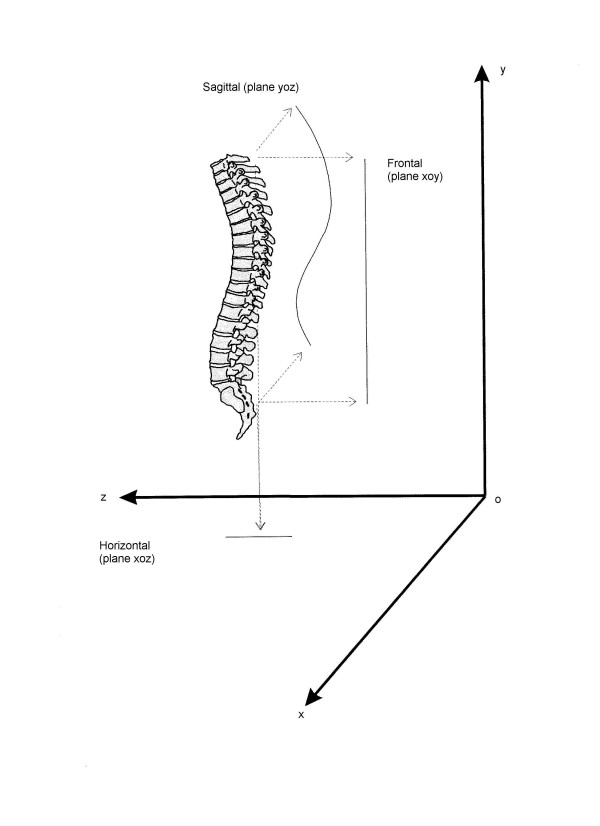
3D representation of an anatomically normal spine according to White and Panjabi (25), and its projections in the three spatial planes – according to the spinal Top View. The normal spine is straight in the frontal plane (xoy), while the sagittal (yoz) physiological curves (kyphosis and lordosis) make it appear, in the Top View (Horizontal – xoz), as a straight line and not as a point, as it would have been in case of absence of sagittal curves. Drawing the TopView from C7 to S1 in a normal situationwould make appear a line first moving backward from C7 to the apex of kyphosis, then forward to the apex of lordosis, and finally backward to the starting point (by definition, in the spinal Top View there is vertical coincidence of C7 and S1) in the middle of the graph.

The Cartesian reference system (Figure 1, 2), on to which the graphic representation of the spine of each subject is projected, is obtained rotating the global reference system to make it a spinal reference system. This is true when the vertical and spinal (C7-S1) axes are coincident, and the center of the reference system is coincident with S1. Measurements are expressed in millimetres.

**Figure 3:**
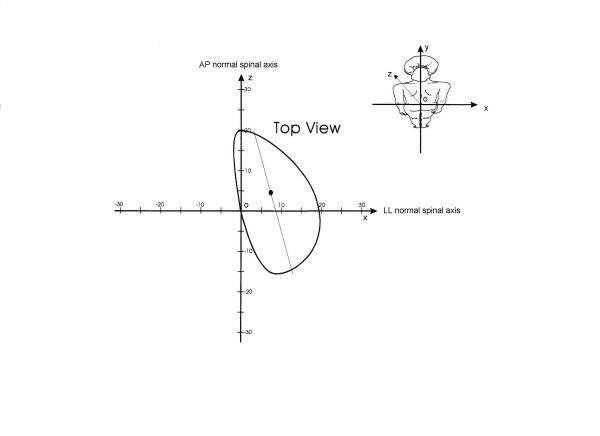
Spinal Top View of a pathological spine with indication of the graphic elements used for the Cartesian reference System. The graphic representation is of the top-back of the examined subject, so that right and front of the graphic correspond to the real right and front. The middle of the Cartesian axes is represented by the line joining C7-S1, i.e. the vertical axis oy that becomes a point in the horizontal xoz plane constituting the Top View. The ordinate (ox) is the latero-lateral (LL) normal spinal axis that has been defined as the line passing through S1 (o) and parallel to the anterior-superior iliac spines. The abscissa (oz) is the antero-posterior (AP) normal spinal axis, that is the line orthogonal (at 90°) to the previous one passing through S1 (o): this line corresponds to the AP body and pelvis axes, as well as to that of the spine in the theoretical normal model of Figure 2.

In this study we refer to a concept of spine anatomical normality, derived from the vertebral column model proposed by White and Panjabi [33]: for a normal subject (Figure 2), the Cartesian reference system should coincide with the laboratory one. The representation regards the top-back of the examined subject, so that right and front of the graph correspond to the real right and front (Figure 3).

**Figure 4:**
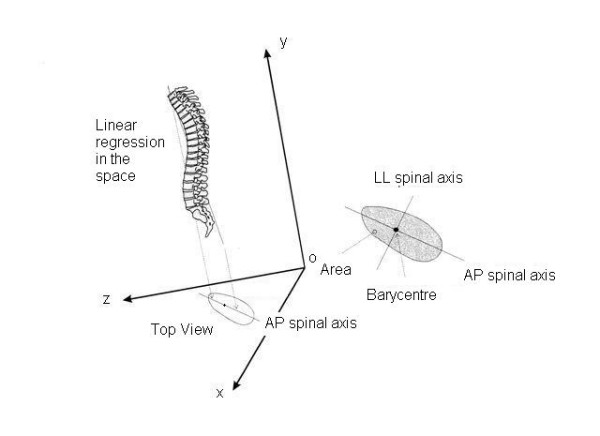
Graphic representation of a pathological spine according to the spinal Top View and its correspondence with the 3D real spine. Spinal Top View: projection on to the horizontal plane of the spine morphology. Barycentre: barycentre of the points reconstructing the spine projected in the horizontal plane. Antero-posterior (AP) spinal axis: projection on to the horizontal plane of the 3D linear regression of the markers on the spinal apophyses of the spine. Latero-lateral (LL) spinal axis: the axis orthogonal (90°) to the AP spinal axis passing through the barycentre. Area: the surface area limited in the horizontal plane by the Top View.

The "quasi-3D" graphic representation of the spine, according to the spinal Top View, includes (Figure 3 and 4):

• the AP (abscissa) and LL (ordinate) spinal axis defined as normal according to the White and Panjabi vertebral column model, that constitutes the Cartesian reference system; the intersection of these axes is the line joining C7-S1, that is the vertical axis of the 3D representation in a spinal reference system; the AP and LL normal spinal axes coincide with the body axes;

• the Top View, i.e. the area resulting from the projection of the spine on the horizontal plane;

• the barycentre, i.e. the barycentre (centre of gravity) of the Top View;

• the AP spinal axis, i.e. the regression line of the Top View;

• the LL spinal axis, i.e. the axis orthogonal (90°) to the AP spinal axis passing through the barycentre;

• the area, i.e. the surface area of the Top View.

### Data analysis

For data processing we used our own software written in C++. For statistical analysis we used SAS JMP 6.0 for Windows. To compare the population we applied the chi squared test and statistical significance was set at P < 0.05. We used the Shapiro-Wilk W test to verify the normal distribution. To establish normative data, we considered the upper and lower 95% quantiles obtained in the normal sample.

## Results

### Direction

#### Observation

**Figure 5:**
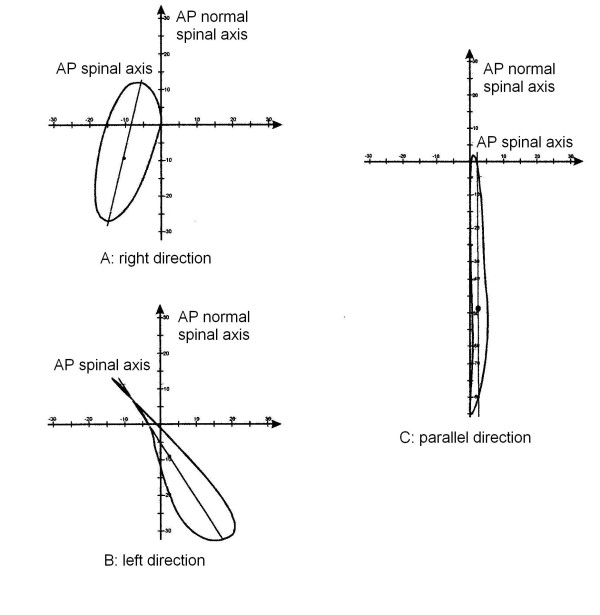
Examples of patients with different Directions. The Direction classificatory parameter is the angle between the AP spinal axis and the AP normal spinal axis (abscissa); it was defined as Direction because it is as if the pathological spine had changed its normal postero-anterior direction with respect to the pelvis, rotating clockwise or anticlockwise. A: right Direction; B: left Direction; C: parallel Direction. For clinical and 3 DEMO complete data of these patients see Appendix [see Additional file 1].

we verified that it was possible to group the curves according to the orientation of the AP spinal axis with respect to the AP normal spinal axis (Figure 5).

#### Definition

the Direction classificatory parameter is the angle between the AP spinal axis and the AP normal spinal axis; it was defined as Direction because it is as if the pathological spine had changed its normal postero-anterior direction with respect to the pelvis, rotating clockwise or anticlockwise.

#### Calculation elements

Direction is equal to the angle between the AP spinal axis and the AP normal spinal axis. A positive sign means a clockwise rotation of the AP spinal axis.

#### Classificatory options

• right (Figure 5A): the AP spinal axis is rotated, in a postero-anterior direction, to the right with respect to the AP normal spinal axis;

• left (Figure 5B): the AP spinal axis is rotated, in a postero-anterior direction, to the left with respect to the AP normal spinal axis;

• parallel (Figure 5C): rotation of the spinal axis is between the normal limits.

#### Norms

95% quantiles in the normal population gave a range of normality – from 9.1 to the left to/13.1° to the right.

#### Numerical results (Table 2)

**Table 2:** Division of studied population according to the classificatory 3-DEMO parameter "Direction".

Classification	Limits	Scoliosis	Hyperkyphosis	Scoliosis & Hyperkyphosis	Normals
Left	Angle < -9.1	50.0%	0.0%	25.0%	0.0%
Right	Angle > 13.1	9.8%	8.7%	50.0%	5.0%
Parallel	-9.1 < angle < 13.1	40.2%	91.3%	25.0%	95.0%
Chi-Square test		P < 0.05			

The Direction classificatory parameter is the angle between the AP spinal axis and the AP normal spinal axis (abscissa); it was defined as Direction because it is as if the pathological spine had changed its normal postero-anterior direction with respect to the pelvis, rotating clockwise or anticlockwise.

the normal Direction of the spine is slightly toward right (mean 2.0°, 2.4° and 3.0° in the three evaluations performed in the normal sample); all normal spines who did not have a parallel Direction had a right one. On the contrary, in scoliosis population the most frequent Direction was left (50%), but there was also a large percentage of parallel results (40%); as expected, the most frequent Direction in the hyperkyphosis group was parallel (91%). A statistically significant difference emerged between the groups (P < 0.05).

### Shift

#### Observation

**Figure 6:**
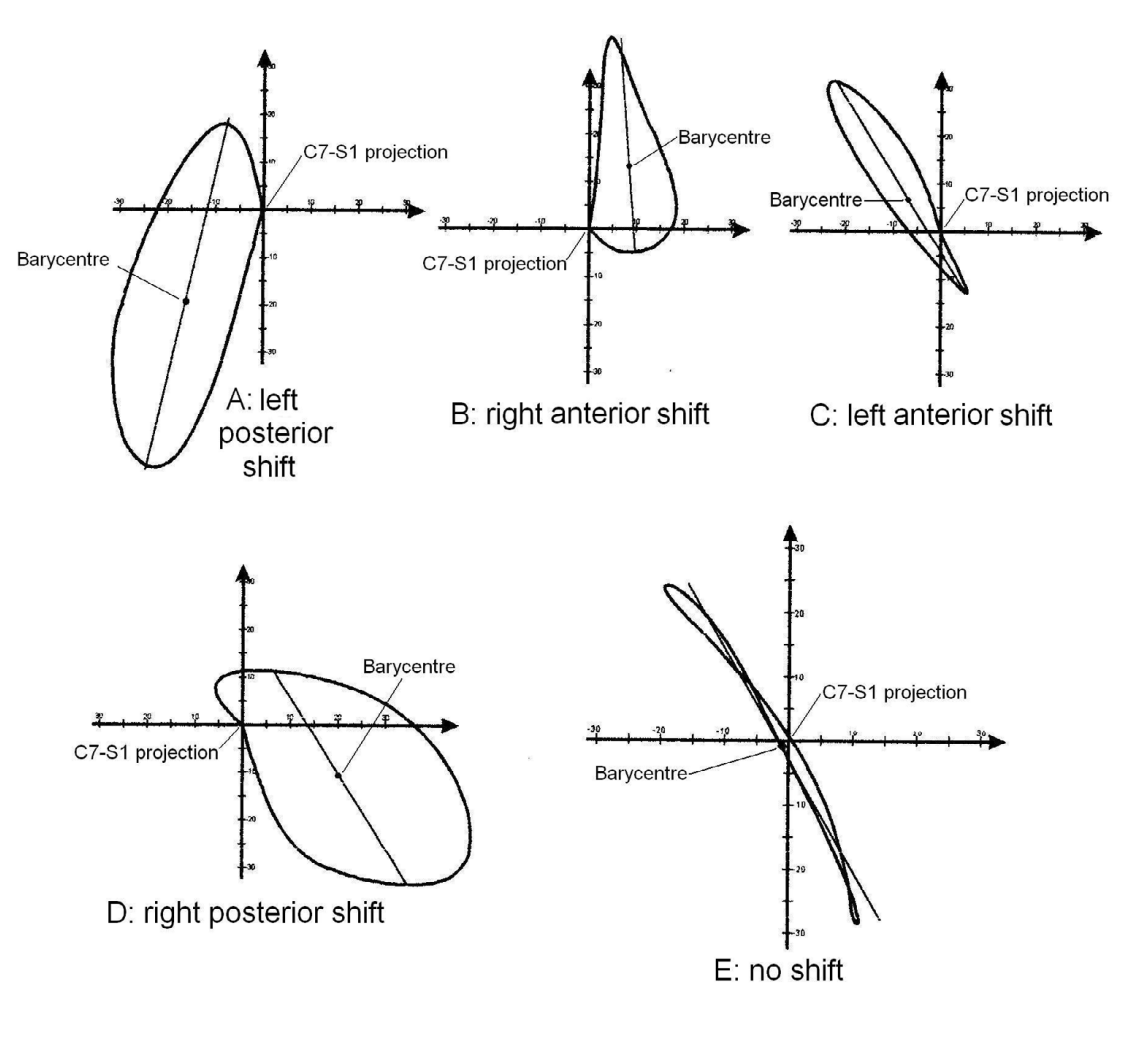
Examples of different Shifts. The Shift classificatory parameter is the displacement of the barycentre of the Top View with respect to the spinal normal axis; it was defined as Shift because it is as if the pathological spine had changed its position with respect to the pelvis, "shifting" away from the vertical C7-S1 axis. A: Left Posterior Shift; B: Right Anterior Shift; C: Left Anterior Shift; D: Left Posterior Shift; E: no shift. For clinical and 3 DEMO complete data of these patients see Appendix [see Additional file 1].

we verified that it was possible to group the curves according to a different projection of the spinal curve with respect to the normal spine here identified through the spinal reference system (Figure 6).

#### Definition

the Shift classificatory parameter is the displacement of the barycentre of the Top View with respect to the spinal normal vertical axis; it was defined as Shift because it is as if the pathological spine had changed its position with respect to the pelvis, "shifting" away from the vertical C7-S1 axis.

#### Calculation elements

Shift is equal to the co-ordinates of the barycentre in the plane of the spinal Top View.

#### Classificatory options

• Right Shifted (Figures 6B,D): the barycentre is located on the right side with respect to the AP normal spinal axis;

• Left Shifted (Figures 6A,C): the barycentre is located on the left side with respect to the AP normal spinal axis;

• Anterior Shift (B,C): the barycentre is located to the front with respect to the LL normal spinal axis;

• Posterior Shift (Figures 6A,D): the barycentre is located to the back with respect to the LL normal spinal axis;

• No shift (Figure 6E): the displacement of the barycentre of the Top View with respect to the spinal axis is between the normal limits.

#### Norms

95% quantiles in the normal population gave these ranges of normality: Lateral Shift: 8.0 mm. to the right, 4.1 to the left.; Sagittal Shift: 26.4 mm. posteriorly, 4.6 anteriorly.

#### Numerical results (Table 3)

**Table 3:** Division of studied population according to the classificatory 3-DEMO parameter "Shift".

Classification	Limits	Scoliosis	Hyperkyphosis	Scoliosis & Hyperkyphosis	Normals
Right Shift	x < -8.0	9.8%	4.3%	50.0%	5.0%
Left Shift	x > 4.1	49.2%	8.7%	25.0%	0.0%
Frontally no Shift	-8.0 < x < 4.1	41.0%	87.0%	25.0%	95.0%
Chi-Square test		P < 0.05			
Anterior Shift	x < -26.4	0.0%	0.0%	0.0%	0.0%
Posterior Shift	x > 4.6	44.3%	100.0%	75.0%	5.0%
Sagittally no Shift	-26.4 < x < 4.6	55.7%	0.0%	25.0%	95.0%
Chi-Square test		P < 0.05			
Posterior Left Shift		17.2%	8,7%	0.0%	0.0%
Posterior Right Shift		6.6%	4.3%	50.0%	5.0%
Posterior Shift only		20.5%	87.0%	25.0%	0.0%
Left Shift only		32.0%	0.0%	25.0%	0.0%
Right Shift only		3.3%	0.0%	0.0%	0.0%
No Shift		20.5%	0.0%	0.0%	95.0%

The Shift classificatory parameter is the displacement of the barycentre of the Top View with respect to the spinal normal axis; it was defined as Shift because it is as if the pathological spine had changed its position with respect to the pelvis, "shifting" away from the vertical C7-S1 axis.

the normal lateral Shift of the spine is toward the right (means 2.2, 2.1 and 1.9 mm respectively in the three evaluations performed in the normal sample), as well as on the sagittal plane is posterior (means 13.0, 13.1 and 10.9 mm respectively); if shifted, all normal spines are posteriorly on the right. On the contrary, in scoliosis population the most frequent lateral Shift was left (49%), while the majority of the curves was found not to be shifted in the sagittal plane (56%); in the hyperkyphosis group the most frequent lateral Shift was right (87%) while all curves were found to be backward shifted in the sagittal plane. Statistically significant differences emerged between scoliosis and hyperkyphosis groups (LL Shift: χ^2 ^test: 13.57, P < 0.05; AP Shift: χ^2 ^test: 23.25, P < 0.05).

### Phase

#### Observation

**Figure 7:**
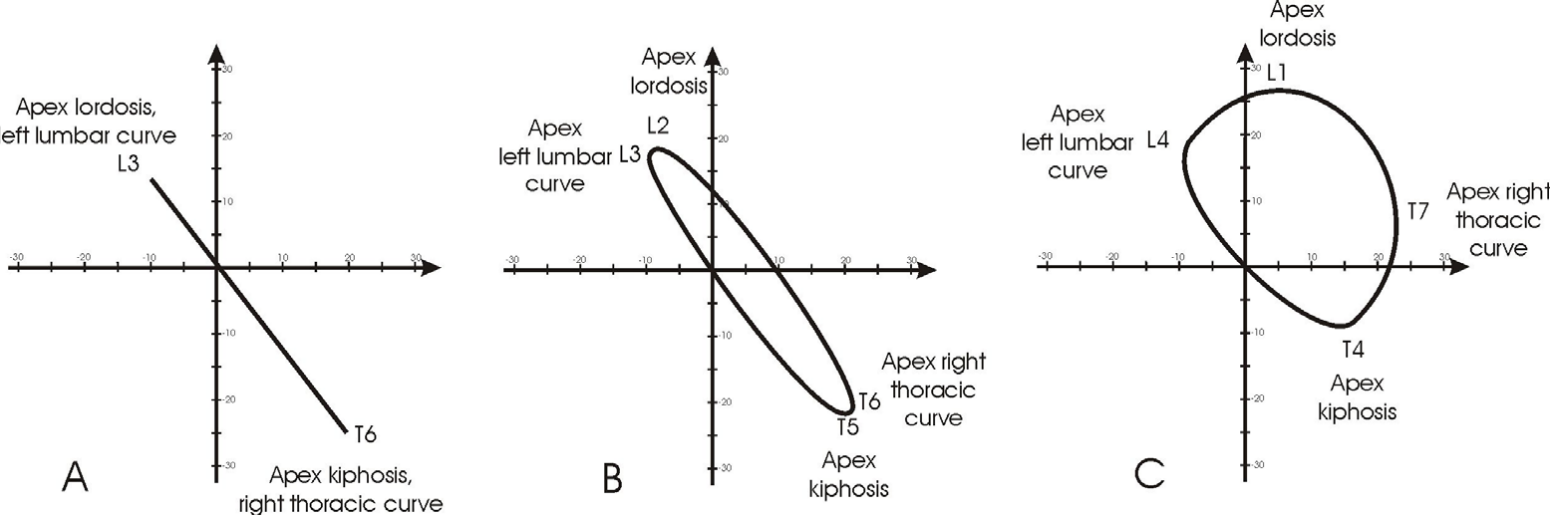
In electronic signaling, phase is an expression of relativemovements between two waves: when they have the apexes coincident they are "in phase". Looking at the curves of scoliosis in the frontal plane and kyphosis/lordosis in the sagittal, they can be imagined as waves: by analogy, we called the relationship between these two "waves", one physiological, the other pathological, Phase, because it takes into account the reciprocal relationship (localization and morphology) among them. In this Figure three different clinical situations in which the classical radiographic curves can be defined "in phase" (isophasic) or not "in phase" (anisophasic) have been simulated. In these hypothetical clinical cases all scoliosis curves have an amplitude of 30° Cobb and all sagittal curves of 40°. A. Isophasic scoliosis: double frontal curve with identical apex vertebrae in the frontal and sagittal planes; the right thoracic curve and kyphosis have both the apex in T6, while the left lumbar and lordosis in L3. B. First anisophasic scoliosis: double frontal curve with a slight difference between the apex vertebrae in the frontal and sagittal planes; the right thoracic curve has the apex in T6 while kyphosis in T5, the left lumbar in L3 and lordosis in L2. C. Second anisophasic scoliosis: double frontal curve with an important difference between the apex vertebrae in the frontal and sagittal planes; the right thoracic curve has the apex in T7 while kyphosis in T4, the left lumbar in L4 and lordosis in L1.

we verified that it was possible to group the curves according to completely different evolutions of the spine in space, that made some curves similar to circles around the barycentre and some others similar to lines (Figure 7).

#### Definition

**Figure 8:**
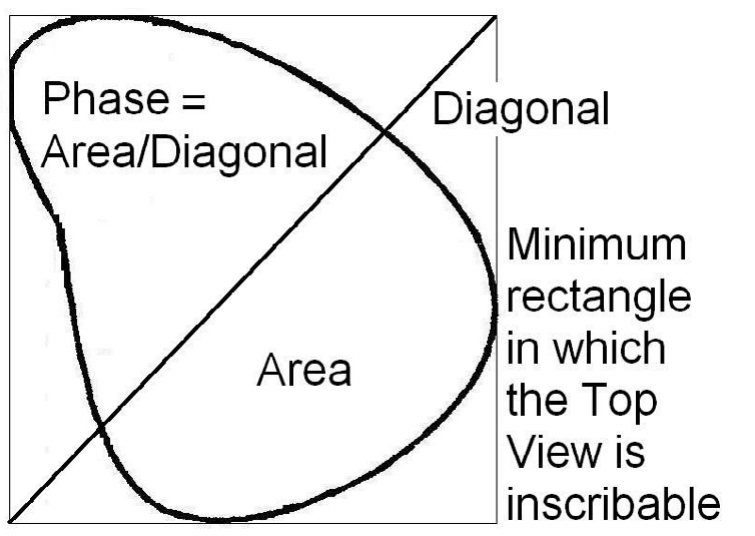
The classificatory parameter defined Phase is the Top View graphical representation of the 3D spatial evolution of the curve as reported in Figure 4. This parameter is obtained dividing the spinal area for the diagonal of the minimum rectangle in which the Top View is inscribable. This allows to verify mathematically how much is "open" the curve considered: the highest value is reached with a circle (anisophasic curve, according to our definition), the lowest with a line (isophasic curve, according to our definition).

the classificatory parameter defined Phase is obtained dividing the Top View area for the diagonal of the minimum rectangle in which the Top View is inscribable (Figure 8); in practice, Phase is a measure of the 3D spatial evolution of the curve; this feature was defined as Phase because it takes into account the reciprocal relationship (localization and morphology) among spinal curves projected in the frontal and sagittal planes, and usually seen at the radiographic examination; the pathological spine has new curves in the frontal plane, that may or may not be "in phase" with the physiological curves in the sagittal plane (Figure 7).

#### Calculation elements

Phase is evaluated by dividing the area for the diagonal of the minimum rectangle in which the Top View is inscribable (Figure 8).

#### Classificatory options

**Figure 9:**
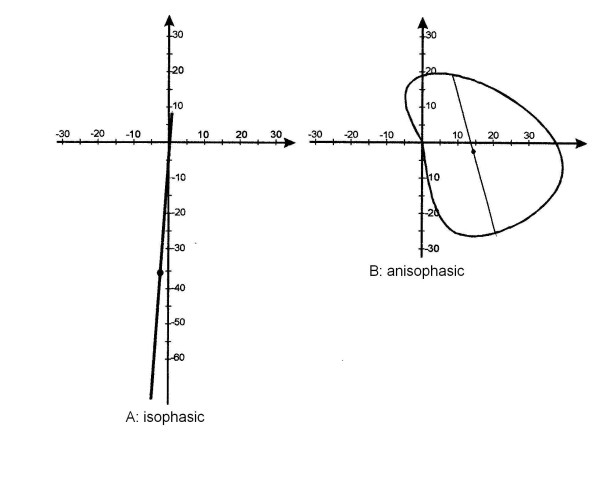
Examples of curves with different Phases. The classificatory parameter defined Phase is obtained dividing the spinal area for the diagonal of the minimum rectangle in which the Top View is inscribable; in practice, Phase is the Top View graphical representation of the 3D spatial evolution of the curve. A: Isophasic; B: Anisophasic. For clinical and 3 DEMO complete data of these patients see Appendix [see Additional file 1].

• isophasic (Figure 9A): the development of the curve gives a low area/diagonal value; the end and apex vertebrae of the frontal and sagittal curves are close to or coincide with one another (Figure 7A); it is as if the pathological spine had simply become elongated (hyperkyphosis, hyperlordosis) or had rotated around the barycentre (scoliosis) with respect to the pelvis and the body, without changing its shape in a complete way;

• anisophasic (Figure 9B): the development of the curve gives a high area/diagonal value; it is as if the pathological spine had completely changed its appearance, enlarging, like a circle, its projection in the horizontal plane; there is no coincidence between the end and apex vertebrae of the curves in the frontal and sagittal planes (Figure 7B).

#### Norms

95% quantiles in the normal population gave the limit of 8.1 mm.: over this value the curve is anisophasic.

#### Numerical results (Table 4)

**Table 4:** Division of studied population according to the classificatory 3-DEMO parameter "Phase".

Classification	Limits	Scoliosis	Hyperkyphosis	Scoliosis & Hyperkyphosis	Normals
Anisophasic	Phase > 8.1	58.2%	0.0%	50.0%	3.6%
Isophasic	Phase < 8.1	41.8%	100.0%	50.0%	96.4%
Chi-square test		P < 0.05			

The classificatory parameter defined Phase is obtained dividing the spinal area for the diagonal of the minimum rectangle in which the Top View is inscribable; in practice, Phase is the Top View graphical representation of the 3D spatial evolution of the curve.

normally the spine is isophasic (mean 4.0, 3.9 and 4.0 mm. in the three evaluations performed in the normal sample), as well as in the hyperkyphosis group, while in scoliosis population the most frequent Phase was anisophasic (58%). A statistically significant difference emerged among groups (P < 0.05).

## Discussion

The main results obtained in our study can be summarized as follows:

• there exists the possibility of obtaining a 3D morphological classification (3-DEMO) using the spinal Top View of the spine (which proved to be more useful than the global one);

• in the Top View some pathological spines show a rotation of the AP spinal axis with respect to the AP normal spinal axis, that we called "Direction";

• in the Top View some pathological spines show a displacement of the spinal barycentre with respect to the normal spinal axis, that we called "Shift";

• in the Top View all spines show an evolution in space that makes some curves similar to circles around the barycentre and others similar to lines; this morphological parameter was called "Phase";

• as expected [33], the normal spine is isophasic, but there are some deviations from the normal model (Figure 2), even if they are of low degree, with a prevalence of right Direction and Shift.

We verified that this projection could be extremely useful for grouping different cases of patients with spinal deformities according to their morphological characteristics. The Top View has been widely used in the past [1,2,24-30] because of its capacity to integrate information usually derived from the AP and LL projections, but its intelligibility has always been limited by the adopted reference system (global instead of spinal). By adopting the global reference system, the spine is projected in the horizontal plane in relation to a reference axis which corresponds to the line of gravity and not to a spinal vertical line derived from spinal landmarks. Kohashi et al. [34] published a study in which a spinal Top View is proposed. In this case, the view was obtained from two stereoscopic radiographs, by feeding into a personal computer the data regarding the position of vertebrae centroids identified on radiographic images. The authors used the Top View in order to obtain information having prognostic value, but failed to discuss its validity as an auxiliary plane which might help to further our understanding of scoliotic deformity three-dimensionality.

Direction is defined as a rotation of the AP spinal axis with respect to the AP normal spinal axis. In conditions of anatomical normality [33], the Direction of the AP spinal axis should be orthogonal to the pelvis and should coincide with the AP normal spinal axis that corresponds to the AP bodily axis. However, according to our normal sample, there is a slight rotation to the right of almost 2°. This parameter is 3D in the sense that it allows the spinal curve to be defined as right or left regardless of curves localization in AP radiographs. It is a rotation which, resulting from a pathological orientation assumed by the vertebral column with respect to the pelvis, involves the whole spine. Any change of Direction in the spinal axis is obviously pathological. Such a change should generally be present in a scoliosis population, as it implies a 3D deformity, but not in a hyperkyphosis sample. The statistically significant difference which emerged among our groups confirms this hypothesis. The hyperkyphosis population had three-dimensionally the same behaviour as the normal one. So far it is not possible to present any definitive correspondence between such a pathological axis and elements already reported in the literature, such as the maximum curvature plane [35] (the plane in which spine projection shows the maximum deformity) and/or the rotation between shoulders and pelvis [30]. Although it is quite probable that a relationship does exist between this parameter and other literature data, it offers, in our view, the possibility of combining in a useful, understandable and strictly 3D spinal-related manner the information obtained using other methods.

Shift is defined as a displacement of the spinal barycentre with respect to the C7-S1 vertical line and the pelvis. In anatomically normal conditions, no lateral Shift should be present even if in our own normal sample we found a slight right shift of less then half centimetre. We also had a posterior Shift of almost 1 centimetre from the zero which coincide with S1. This parameter is 3D because it relates to a single point which integrates the information relative to spine behaviour in space and to its displacement with respect to its natural basis, i.e., the pelvis and S1. A high displacement of the spinal barycentre is obviously pathological as, conceptually, it indicates an asymmetry in the positioning of the vertical central axis of spine projection. Because it implies a frontal curve, the variation along the LL spinal axis should be mainly present in a scoliosis population and far less in a hyperkyphosis sample. These statements are confirmed by our data; scoliosis population mainly shows a displacement to the left, but further studies are needed in order to elucidate these findings. The displacement along the AP spinal axis is interesting: on the basis of our current knowledge of the pathologies, we can hypothesize that a scoliotic deformity could show a forward localization of the barycentre, because scoliosis is known to drive the spine forward [36]; the opposite could be true for hyperkyphosis. Only the latter was found to be true, while all pathologically sagittally oriented scoliotic spines were posteriorly Shifted (44.3%). An element which is apparently similar to the Shift, usually highlighted both during the clinical and the radiographic examination, is described in the literature in the frontal plane by the imbalance between C7 and S1 [35,37]. As a matter of fact, this parameter deeply differs from the Shift as, in our spinal Top View, C7 is, by definition, located on the axis of symmetry. Other everyday clinical findings, sometime discussed between specialists, but not published in indexed literature, such as the displacement of the radiological transitional point between thoracic and lumbar/thoracolumbar curve in relation with the central sacral line, or the shift of the rib cage with respect to the pelvis, or even the shift of the stable vertebra, could correlate with Shift as here defined: future studies could address these points.

**Figure 10:**
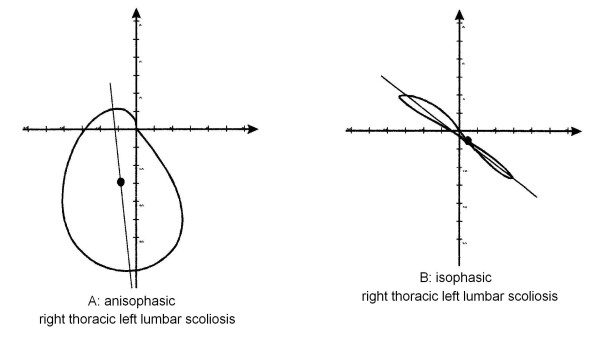
Graphical representation of the top view in two patients with the same Ponseti, King and Lenke classification, very similar Cobb values, completely different 3D behaviour and consequently 3-DEMO classification; for clinical and 3 DEMO complete data of these patients see Appendix [see Additional file 1].

Phase is defined as a description of the evolution in space that makes some curves similar to circles around the barycentre and some others similar to lines. The name derives from the relationship between the spinal curves projected in the frontal and sagittal planes that together give rise to the appearance of the Top View curve. When no curves are present in the frontal plane (anatomical normality), the Top View must be isophasic. This is not totally true, but the Phase value is very low in normals. This is a real, entirely 3D element and paradoxically the used name, which derives from our habit of viewing the spine in two dimensions (AP and LL) and which coherently describes what happens, is not so coherent with 3D reality and is less authentic than the phenomenon itself. As far as we know, in the literature there are no descriptions of this or of similar elements, and much remains to be elucidated through further research. In particular, there is the question of what is the real nature of this 3D space occupation that some curves show. If a scoliotic patient does not have an alteration of Phase, he/she must not have a Parallel Direction (both elements can obviously be changed together, but without the modification of one of them there cannot be a scoliosis, because there is no curve in the frontal plane) (Figures 8 and 10). Is it possible to hypothesize differences of pathogenesis, treatment, prognosis according to the presence of Phase?

The 3-DEMO classification could represent a basic innovation for the analysis of curve morphology, particularly in case of a 3D deformity such as scoliosis, as this analysis allows to draw a distinction between patients who, on the basis of traditional classifications, appear to be the exactly alike. Figure 10 illustrates the Top View behavior of two patients who were deemed to show the same deformity. According to traditional morphological classificatory parameters, they have the same Ponseti diagnosis, as well as King and Lenke ones; they have similar Cobb degrees in AP and LL projections (+/- 1° and 7°). They differs only slightly as regards the position of the end and apex vertebrae. According to the quasi-3D classificatory parameters, subject A has a curve characterized by Phase, backward and slightly left shifted, not rotated: it is as if the spine had simply greatly enlarged the 3D space that it occupies; subject B has a curve characterized by Direction, isophasic like a normal spine, not shifted: it is as if the spine had somehow maintained the behaviour of a normal spine, simply rotating a lot around the barycentre. On the basis of what it is possible to see, the only traditional morphological element able to explain this result is the difference between the end and apex vertebrae, which justifies a difference of Phase. This is an explanation of a 3D phenomenon (shown by our analysis) which makes use of 2D terminology.

## Conclusion

Through this work we propose the 3-DEMO (Three-dimensional, Easy Morphological) classification of vertebral deformities from a clinical point of view. The word "Easy" refers to the final classification, because the existing 3-D classifications are not. We think that the concepts of Direction and Shift are easy, while Phase it is so graphically, but also theoretically, once understood. The technological system (AUSCAN) used to develop the classification was unavoidably a complex and not every-day clinical usage one, because we needed to have a three-dimensional representation of many curves to look at and to develop an insight (such as this one) to be translated in the next future on everyday practice. In fact, we are already working on x-rays and clinical measurements to obtain the same results without the AUSCAN System (this will be presented in a future paper). The novelty of this classification is the application of the quasi-three-dimensionality concept to spine visualization in an auxiliary plane (horizontal plane), the Top View. The chosen spinal analysis [6] was the only one which allowed both a reduction in the inherent variability of the classic Top View (global) and the possibility of achieving an isolated view of the spine. We are not, at the current stage, able to fully appreciate what benefits might derive from the 3-DEMO classification. Nevertheless we believe that there is a need to achieve at least an initial codification of the third dimension and that such a codification could, through its wide clinical application, come to be understood in all its implications and prove or not to be of value.

## Supplementary Material

Additional file 1 Parte 1 Appendix. in this file all clinical and 3-DEMO data and classification of patients represented in the figures of this paper are reported Click here for file
