# Supplementary material for: Three-dimensional easy morphological (3-DEMO) classification of scoliosis, part I
Source: Scoliosis. 2006 Dec 5;1:20. doi: 10.1186/1748-7161-1-20 (PMC1764765; doi:10.1186/1748-7161-1-20)
Supplement: Additional file 1 — Parte 1 Appendix. in this file all clinical and 3-DEMO data and classification of patients represented in the figures of this paper are reported [file 1748-7161-1-20-S1.doc]

# Appendix

We used the parameters defined in the paper to derive a real codification of each single curve. As shown in the table, which lists the 3-DEMO classification of the curves that are reported in the figures, this is done by proposing Direction, Phase and Shift parameters (in that order). We decided to omit all terms that characterize an anatomically normal spine: "parallel" for the Direction, "isophasic" for the Phase, "not shifted" for the Shift, both in frontal and sagittal planes.

| **Figure** | **Patient** | **Classification of Ponseti** | **Scoliosis** | **Kyphosis** | **Lordosis** | **3-DEMO Classification** |
| --- | --- | --- | --- | --- | --- | --- |
| 5a | Female | Left Thoracic | D6-D12 65° | 51° | 68° | Right direction, anisophasic, backward/left shifted |
|  |  | Right Lumbar | D12-L4 45° |  |  |  |
|  |  | Hyperkyphosis, hyperlordosis |  |  |  |  |
| 5b | Female | Right Thoracic | D5-D11 36° | 38° | 32° | Left direction, anisophasic, backward/right shifted |
|  |  | Left Lumbar | D11-L4 18° |  |  |  |
|  |  | Hyperlordosis |  |  |  |  |
| 5c | Female | Hyperkyphosis | D4-D9 10° | 53° | 41° | Backward/right shifted curve |
|  |  | Right Thoracic |  |  |  |  |
| 6a | Male | Hyperkyphosis | D10-L3 17° | 61° | 50° | Right direction, anisophasic, backward/left shifted |
|  |  | Left Thoracolumbar |  |  |  |  |
| 6b | Female | Right Thoracic | D10-L1 40° | 13° | 32° | Anisophasic, /right shifted curve |
| 6c | Female | Right Thoracic, | D2-D9 32° | 31° | 45° | Left direction, anisophasic, /left shifted |
|  |  | Left Thoracolumbar | D9-L4 26° |  |  |  |
| 6d | Female | Right Thoracic | D2-D9 32° | 31° | 45° | Left direction, anisophasic, backward/right shifted |
|  |  | Left Lumbar | D9-L4 26° |  |  |  |
| 6e | Female | Right Thoracic | D2-D10 29° | 22° | 53° | Left direction |
|  |  | Left Lumbar | D10-L4 41° |  |  |  |
|  |  | Hyperlordosis |  |  |  |  |
| 9a | Male | Hyperkyphosis |  | 61° | 45° | Backward/left shifted direction |
|  |  | Left Thoracic | D1-L1 15° |  |  |  |
|  |  | Right lumbar | L1-L5 8° |  |  |  |
| 9b | Female | Left Thoracic | D1-D7 41° | 17° | 37° | Left direction, anisophasic, right shifted |
|  |  | Right Thoracolumbar | D7-L2 64° |  |  |  |
| 10a | Female | Right Thoracic | D3-D9 38° | 39° | 41° | Anisophasic, backward/left shifted direction |
|  |  | Left Lumbar | D9-L3 42° |  |  |  |
| 10b | Female | Right Thoracic | D5-D12 36° | 38° | 44° | Left direction |
|  |  | Left Lumbar | D12-L4 40° |  |  |  |
